# Metabolite Profiling of Methanolic Extract of *Gardenia jaminoides* by LC-MS/MS and GC-MS and Its Anti-Diabetic, and Anti-Oxidant Activities

**DOI:** 10.3390/ph14020102

**Published:** 2021-01-28

**Authors:** Kandasamy Saravanakumar, SeonJu Park, Anbazhagan Sathiyaseelan, Kil-Nam Kim, Su-Hyeon Cho, Arokia Vijaya Anand Mariadoss, Myeong-Hyeon Wang

**Affiliations:** 1 Department of Bio-Health Convergence, Kangwon National University, Chuncheon 24341, Korea; saravana732@kangwon.ac.kr (K.S.); sathiyaseelan.bio@gmail.com (A.S.); mavijaibt@gmail.com (A.V.A.M.); 2 Chuncheon Center, Korea Basic Science Institute (KBSI), Chuncheon 24341, Korea; sjp19@kbsi.re.kr (S.P.); knkim@kbsi.re.kr (K.-N.K.); chosh93@kbsi.re.kr (S.-H.C.); 3 Department of Medical Biomaterials Engineering, College of Biomedical Sciences, Kangwon National University, Chuncheon 24341, Korea

**Keywords:** *Gardenia jasminoides* Ellis, anti-diabetic activity, LC-MS/MS, GC-MS, anti-oxidant

## Abstract

In this study, the methanolic extract from seeds of *Gardenia jasminoides* exhibited strong antioxidant and enzyme inhibition activities with less toxicity to NIH3T3 and HepG2 cells at the concentration of 100 µg/mL. The antioxidant activities (DPPH and ABTS), α-amylase, and α-glucosidase inhibition activities were found higher in methanolic extract (MeOH-E) than H_2_O extract. Besides, 9.82 ± 0.62 µg and 6.42 ± 0.26 µg of MeOH-E were equivalent to 1 µg ascorbic acid for ABTS and DPPH scavenging, respectively while 9.02 ± 0.25 µg and 6.52 ± 0.15 µg of MeOH-E were equivalent to 1 µg of acarbose for inhibition of α-amylase and α-glucosidase respectively. Moreover, the cell assay revealed that the addition of MeOH-E (12.5 µg/mL) increased about 37% of glucose uptake in insulin resistant (IR) HepG2 as compared to untreated IR HepG2 cells. The LC- MS/MS and GC-MS analysis of MeOH-E revealed a total of 54 compounds including terpenoids, glycosides, fatty acid, phenolic acid derivatives. Among the identified compounds, chlorogenic acid and jasminoside A were found promising for anti-diabetic activity revealed by molecular docking study and these molecules are deserving further purification and molecular analysis.

## 1. Introduction

Diabetes mellitus (DM) is a commonly detected chronic disorder causing major mortality worldwide. The progression of diabetes in the global population was reported as 9.3% by 2019 and projected to increase about 10.2% by 2030 and 10.9% by 2045 [1]. Metabolic malfunctions such as high elevation of the blood sugar (glucose) levels, oxidative stress and abnormal protein and lipid metabolism all lead to DM [2]. DM is categorized into two types: insulin-dependent type-1 diabetes (T1DM) and non-insulin-dependent type-2 diabetes (T2DM) [3]. Diabetic patients who are not able to secrete insulin are characterized as T1DM [4], while patients with insulin deficiency or insulin resistance in the human metabolic system, less insulin sensitivity or signaling in the liver, skeletal muscles, and adipose tissue are characterized as T2DM [5,6]. The prolonged diabetic symptoms (hyperglycemia, polyphagia, polydipsia, and insulin resistance) trigger multiple disorders such as cardiovascular diseases, renal failure, coronary artery, neurological complications, premature death, and limb amputation [7,8]. The diabetes incidence is higher in urban areas than in rural areas. Up 50% of people do not know that they are affected by diabetes [1].

Enzymes such as α-amylase and α-glucosidase play a vital role in carbohydrate metabolism. α-Amylase catalyzes the conversion of starch into glucose, while α-glucosidase regulates the p53 signaling pathway and the cleavage of glucose from disaccharides [9,10]. Therefore, the intake of foods rich in enzyme (α-amylase and α-glucosidase) inhibitors can beneficially reduce the risk of T2DM. However, some commercially available enzyme inhibitor show side effects. For example, miglitol, voglibose, and acarbose can induce diarrhea, bowel disruption, abdominal distress, and these drugs are also not recommended to patients with gastrointestinal disorders [11]. Therefore, isolation of new α-amylase and α-glucosidase inhibitors from natural resources with less adverse effects can be considered as an alternative to existing enzyme inhibitors. α-Amylase and α-glucosidase inhibitors can be virtually screened using the molecular docking methods. That way an active imine derivative has been reported for the inhibition of these enzymes by targeting the human lysosomal acid-α-glucosidase (PDB: 5NN8) and human pancreatic α-amylase (PDB: 5E0F) [12].

Worldwide about 80% of people use herbal medicines to cure various diseases [13]. Herbal medicines have also received attention in diabetes healthcare. The investigation and isolation of novel compounds from indigenous herbal plants to cure diseases can expand the economic value of the traditional herbal industry. *G. jasminoides* is a shrub belonging to the *Rubiaceae* family and its metabolites have been proved to possess a variety of ethnopharmacological properties [14]. Traditionally *G. jasminoides* has been used as folk medicine, as a functional food and a food colorant in Asian countries [15]. The pigments produced from the ripe fruits of this plant have been used as a natural food colorant. The metabolites of *G. jasminoides* is used as a traditional natural medicine as a diuretic and to cure hemostasis, hypotension (low blood pressure) and to increase blood circulation [14]. Moreover, the pigments are not only used as a food colorant but also applied as beneficial health-promoting agents [16]. The compounds from *G. jasminoides* display promising pharmacological activities that are reviewed in earlier literature [14,17]. For instance, genipin, geniposide, crocin and crocetin isolated from *G. jasminoides* possess antidepressant, antidiabetes, antioxidant and antihypertensive activities [18,19,20,21], which has prompted additional studies to screen and identify metabolites active against T2DM. Therefore, the present study was aimed at investigating the metabolite profile of MeOH-E of *G. jasminoides* by LC-MS/MS, GC-MS and screen its anti-diabetic, and anti-oxidant effects using in vitro cytotoxicity, antioxidant, and enzyme inhibitory assays.

## 2. Results and Discussion

### 2.1. Yield, Total Phenol and Total Flavonoids Contents

The yield of different solvent extracts of seed powder of *G*. *jasminoides* was found to be 2.45% (*w*/*w*) and 1.58% (*w*/*w*) for methanol extract (MeOH-E) and water extract (H_2_O-E), respectively (Table 1). The total phenol and flavonoids are major constituents in secondary metabolites of the plant extracts and they play a vital role in the biological properties of plants [22]. The bioactivities of the plant extracts are strongly correlated with the content of total flavonoids and phenolic substances. The *G*. *jasminoides*-derived pigments are shown to have anti-inflammatory, antioxidant, antibacterial activities with bio-health promoting properties by preventing various disorders [14]. Therefore, the content of total phenol (TPC) and total flavonoids (TFC) in MeOH-E and H_2_O-E was determined and the results are expressed as tannic acid equivalents (TAEs) for TPC while the TFC is presented as quercetin equivalents (QEs). For TPC, 769.47 ± 3.74 µg and 632.15 ± 1.25 µg of tannic acid equivalents to one gram of MeOH-E and H_2_O-E, where the TFC 487.54 ± 1.19 µg and 347.00 ± 2.49 µg of quercetin equivalents to one gram of MeOH-E and H_2_O-E, respectively (Table 1).

### 2.2. Antioxidant Activities

Oxidative stress is a major primary cause of various health disorders. Therefore, screening of antioxidants from plant extracts can be a prime way to isolate novel compound against various chronic and metabolic disorders. 1,2-Diphenyl-1-picrylhydrazyl (DPPH) is a stable free radical known to have a purple color with a strong absorption peak at 517 nm. Antioxidants can scavenge the DPPH by donating electrons [23]. (2,2′-Azino-bis(3-ethylbenzothiazoline-6-sulfonic acid) diammonium salt (ABTS^+^) is a commonly used free radical for antioxidant assays. Mixing of ABTS and potassium persulfate produces the free radical form of the ABTS^+^ which can be scavenged by the addition of synthetic or natural antioxidants [23]. The antioxidant activities of the DPPH and ABTS^+^ varied significantly between the H_2_O-E and MeOH-E (*p* < 0.05). Among the samples, the free radical scavenging activity was found higher in MeOH-E than H_2_O-E in a dose-dependent manner. The free radical scavenging activity of these extracts was compared with a standard to obtain the ascorbic acid equivalents (AAEs). The results revealed that 9.82 ± 0.62 µg of MeOH-E and 13.20 ± 1.25 µg of H_2_O-E were equivalent to 1 µg AAEs for ABTS scavenging. It also varied for the DPPH scavenging with the values of 6.42 ± 0.26 µg for MeOH-E and 9.22 ± 0.81 µg for H_2_O-E, which were equivalent to 1 µg of ascorbic acid (Table 2). Further, the IC_50_ concentration was found to be 120.5 ± 1.09 µg/mL and 262.5 ± 0.18 µg/mL for MeOH-E and H_2_O-E, respectively, for the ABTS^+^ radical scavenging (Table 2). In the case of DPPH radical scavenging, the IC_50_ was found to be 274.9 ± 1.42 µg/mL and 573.1 ± 0.85 µg/mL for MeOH-E and H_2_O-E, respectively (Table 2). Similarly, the methanol extract of *G. volkensii* reportedly shows a moderate DPPH scavenging activity [23]. Moreover, an earlier work reported that the water extract of *G*. *jasminoides* shows a higher DPPH and ABTS^+^ scavenging activity than the ethanol extract. It is also observed from earlier study that the water extract of *G*. *jasminoides* exhibited the IC_50_ values of 0.14 and 0.21 mg/mL for DPPH and ABTS ^+^ scavenging activities respectively [24]. This result indicates a variation between the present work and earlier work for IC_50_ of H_2_O-E, probably due to the differences in the extraction method and sample collection location. The present results indicated that the antioxidant activity was higher in MeOH-E than that in H_2_O-E due to a higher total phenolic and flavonoids content [25]. The present work also found a similar relationship between antioxidant activity and total phenol content of MeOH-E and H_2_O-E, which is in accordance with earlier works [23,25].

### 2.3. Enzyme Inhibitory Activities

The enzymes α-amylase and α-glucosidase are involved in carbohydrate metabolism in the conversion of simple sugars from polysaccharides or disaccharides and also in catalyzing the blood glucose level that results in T2DM hyperglycemia [26]. Therefore, inhibition of these enzymes can control the prevalence of T2DM. Moreover, several studies also reported that screening of these enzyme inhibitors is crucial for the discovery of novel diabetes drugs [27,28]. The present work showed the enzyme (α-amylase and α-glucosidase) inhibitory activity of MeOH-E and H_2_O-E of seed powder of *G*. *jasminoides* (Table 2). Among the two samples, MeOH-E exhibited higher α-amylase and α-glucosidase inhibition activities than H_2_O-E. The 9.02 ± 0.25 µg of MeOH-E and 15.22 ± 0.55 µg of H_2_O-E were equivalent to 1 µg of acarbose for α-amylase inhibition activity (Table 2). In the case of α-glucosidase inhibition, 6.52 ± 0.15 µg of MeOH-E and 12.52 ± 0.61 µg of H_2_O-E were found to equivalent to 1 µg of acarbose (Table 2). The IC50 of MeOH-E were found to be 432.05 ± 0.51 µg/mL and 798.25 ± 0.84 µg/mL for α-amylase and α-glucosidase inhibition activity respectively (Table 2). Among the two samples, MeOH-E showed promising activities of antioxidant and α-amylase and α-glucosidase inhibition. Therefore, MeOH-E was selected further for cell culture experiments.

### 2.4. Cytotoxicity

**Figure 1:**
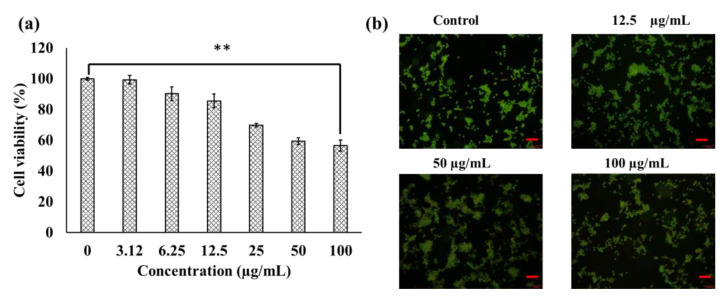
Cytotoxicity of the methanolic extract (MeOH-E) of *G. jasminoides* in NIH3T3 cell line (**a**), AO/EB staining assay (**b**). ** *p* < 0.01 significant. Scale bar 100 µm.

The cytotoxic effects of MeOH-E in a mouse fibroblast (NIH3T3) cell line was determined using a WST assay. The results revealed that MeOH-E at the concentration of ≤12.5 µg/mL did not show any cytotoxicity, while that at >25–100 µg/mL exhibited moderate cytotoxicity in the NIH3T3 cell line (Figure 1a). Similarly, the extract of *G. jasminoides* is reportedly non-toxic to the normal human MCF-10A cell line [29]. Another mouse model experiment confirmed that the pigments derived from *G. jasminoides* are less toxic [30]. Meanwhile, different solvent extracts of *G. jasminoides* have been reported to have promising cytotoxicity towards various cancer cells, including cervical cancer cell line (HeLa), skin malignancy cell line (A375), human non-small cell lung carcinoma cell line (H1299), and breast cancer cell line (MCF-7) [29,31]. However, to ensure the non-cytotoxicity of the MeOH-E in the NIH3T3 cell line the present study applied an acridine orange/ethidium bromide (AO/EB) fluorescent staining assay. This fluorescent method is used to determine the apoptosis-associated changes in cells based on the nucleus damage [32]. The AO/EB staining results indicated no apoptosis cells in the control group, and in the cells treated with 12.5 µg/mL; however, early stage apoptosis cells were observed at 50 µg/mL and 100 µg/mL (Figure 1b). Similarly, the early apoptosis in the osteosarcoma cells was detected by AO/EB staining as indicated by yellow-green and crescent-shaped cells [32].

### 2.5. Effect of MeOH-E on Cell Viability and Glucose Uptake in HepG2 Cell Line

**Figure 2:**
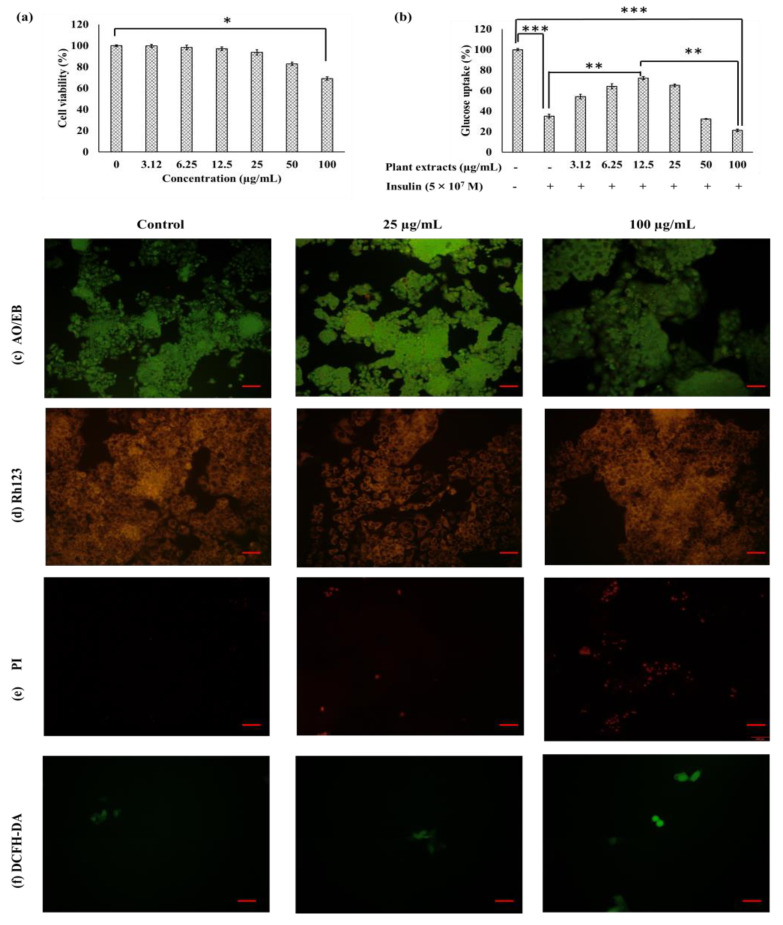
Cytotoxicity of the methanolic extract (MeOH-E) of *G. jasminoides* in insulin-resistant (IR) HepG2 cell line (**a**), glucose uptake (**b**), AO/EB staining assay (**c**), mitochondrial membrane potential (**d**), measurement of nucleus damage by PI (**e**), analysis of the reactive oxygen species generation (**f**). Scale bar 100 µm for C & E, and 50 µm for D & F. * *p* < 0.05, ** *p* < 0.01, *** *p* < 0.001 significant.

MeOH-E did not display significant cytotoxicity on the Hep2 cell line at the concentration of ≤25 µg/mL and only at 100 µg/mL was significant cytotoxicity exhibited (Figure 2a). This revealed the non-toxicity of MeOH-E in the HepG2 cell line at ≤25 µg/mL. Therefore, the effect of MeOH-E treatment in the glucose metabolism was tested by glucose uptake assay in non-insulin resistant and insulin resistant (IR)-HepG2 cell lines. The glucose uptake was found to be higher in the non-IR HepG2 cell line than that in the IR-HepG2 cell line. However, the addition of MeOH-E (12.5 µg/mL) increased ~37% of glucose uptake in IR-HepG2 as compared to untreated IR HepG2 cell line (Figure 2b). This experiment also led to the interesting observation that the treatment above 25 µg/mL of MeOH-E to IR-Hep2 cell line significantly decreased the glucose uptake due to toxicity of the extract (Figure 2b). This is in accordance with an earlier report on ethyl acetate extract of *Physalis alkekengi* in glucose uptake in HepG2 cells [33]. The present work revealed that the treatment of 12.5 µg/mL was optimal for the increased glucose uptake by the IR-HepG2 cell line.

#### Fluorescent Assay

The cytotoxicity of MeOH-E in the HepG2 cell line was measured by fluorescent AO/EB, rhodamine 123 (Rh123), propidium iodide (PI), and 2′-7′dichlorofluorescin diacetate (DCFH-DA) staining assays (Figure 2c–f). The cells were grouped as live cells (light green), apoptosis cells (fluorescent or yellowish, orange), necrosis cells (red) [34]. The MeOH-E at 25 µg/mL and 100 µg/mL) caused slight cytotoxicity for IR-HepG2 cell line as evident by pyknosis and congregated chromatin emitting green or yellow and some red fluorescence while the untreated control cells emitted uniform green fluorescence (Figure 2c). Rh123 staining is adopted to measure the mitochondrial membrane potential (MMP) loss in the HepG2 cell line. Rh123 dye effectively stains with rich MMP and loss of MMP is indicated with the decrease of dye emission [35]. Similarly, the present study observed that the Rh123 was highly emitted in the HepG2 cell line treated with different concentrations of MeOH-E and it indicated less toxicity of extracts (Figure 2d). The PI is an impaired nucleic acid membrane stain used for the detection of dead cells in a cell population [36,37]. The present study observed no PI-stained cells in the untreated control group while the treatment of 25 µg/mL and 100 µg/mL of MeOH-E displayed the dead cells as red-colored (Figure 2e). DCFH-DA staining results indicated that the treatment of MeOH-E (25 µg/mL) did not cause the ROS mediated cytotoxicity while it exhibited slight cytotoxicity in the HepG2 cell line (Figure 2f).

### 2.6. Metabolite Profiling of the MeOH-E of G. jasminoides

To identify the components of the MeOH-E of *G. jasminoides*, we tentatively identified them using two major hyphenated techniques: gas chromatography-mass spectrometry (GC-MS) and liquid chromatography with tandem mass spectrometry (LC-MS/MS), which cover quite different subsets of metabolites. For instance, GC-MS has a preference for volatile metabolites covering primary metabolism including organic and amino acids, sugars, sugar alcohols, and phosphorylated intermediates. In contrast, LC-MS/MS covers mostly polar compounds predominant in secondary metabolites such as phenolics and terpenoids [38,39].

#### 2.6.1. Tentative Identification of Compounds by LC-MS/MS

**Figure 3:**
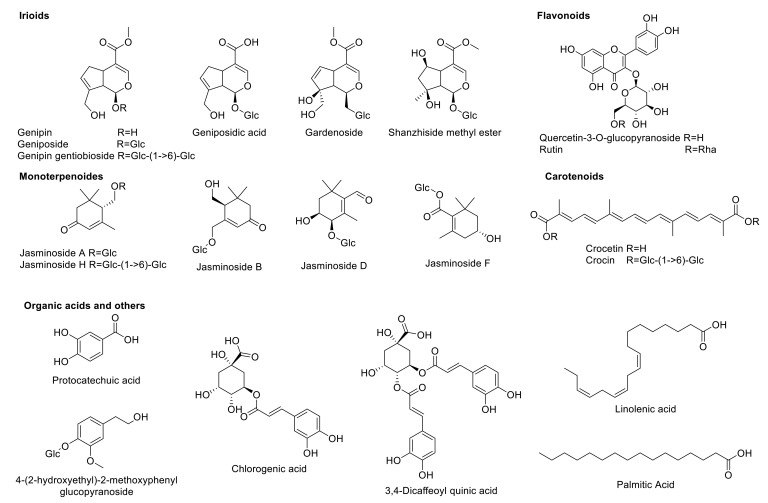
Structures of selected compounds identified by LC-MS/MS.

The compounds present in the MeOH-E were tentatively identified using LC-MS/MS and the TIC chromatogram of metabolic profile of the MeOH-E is shown in the Supplementary Figure S1. The LC-MS/MS analysis revealed the presence of 39 phytochemicals that belonging to various subclasses such as phenolic, flavonoids, terpenes, iridoid glycosides, organic acids, and gardenia carotenoids (Table 3). These compounds were identified based on the *m/z* of molecular ion [M–H]^−^ and interpretation of the MS and MS/MS spectra comparison with the MassLynx V4.1 library (Waters Corporation, Milford, MA, USA). The compounds were identified using the in-house phytochemical library (UNIFI 1.8; Waters) [40,41] and previously reported literature [14,42]. Structures of the selected compounds are presented in Figure 3.

##### Iridoids

The iridoid glycosides are a group of phytochemicals that is commonly present in various families of the plant families including Rubaiaceae [43]. According to our LC-MS/MS analysis, MeOH-E of *G. jasminoides* (Rubiaceae) exhibited compounds such as geniposidic acid (*m*/*z* 373.11), shanzhiside methyl ester (*m*/*z* 405.14), 6β-hydroxygeniposide (*m*/*z* 403.12), gardenoside (*m*/*z* 403.12), genipin gentiobioside (*m*/*z* 549.18), genipin (*m*/*z* 225.07), geniposide (*m*/*z* 387.13), coumaroylgenipin gentiobioside (*m*/*z* 695.21), and feruloylgenipin gentiobioside (*m*/*z* 725.23). Detailed identification information of these compounds such as retention time, formula, observed *m*/*z*, mass error, response and product ion mass are listed in Table 3.

##### Monoterpenoids

The monoterpenes, whether linear (acyclic) or containing rings (bicyclic and monocyclic), belons to a class of terpenes that possess remarkable applications in the food and pharmaceutical industries [55]. *G. jasminoides* was reported to be a rich source of monoterpenoids and a total of 26 monoterpenoids have been reported from *G. jasminoides* [56,57,58,59,60]. The present study identified a total of 13 monoterpenoids from MeOH-E of *G. jasminoides* based on the deprotonated molecular ions observed in the LC-MS/MS analysis. The formulas of identified compounds were as follows: C_10_H_16_O_2_ (*m*/*z* 167.1083), C_10_H_16_O_3_ (*m*/*z* 183.1029), C_12_H_18_O_6_ (*m*/*z* 257.1033), C_13_H_17_NO_5_ (*m*/*z* 266.1039), C_13_H_22_O_3_ (*m*/*z* 225.1495), C_16_H_24_O_7_ (*m*/*z* 327.1446), C_16_H_26_O_7_ (*m*/*z* 329.1606), C_16_H_26_O_8_ (*m*/*z* 345.1599), C_16_H_26_O_9_ (*m*/*z* 361.1506), C_22_H_36_O_12_ (*m*/*z* 491.2123) and C_27_H_34_O_11_ (*m*/*z* 533.2021). The compound names, MS/MS fragmentation patterns, retention times as well as response factors corresponding to each chemical are described in Table 3.

##### Flavonoids

**Figure 4:**
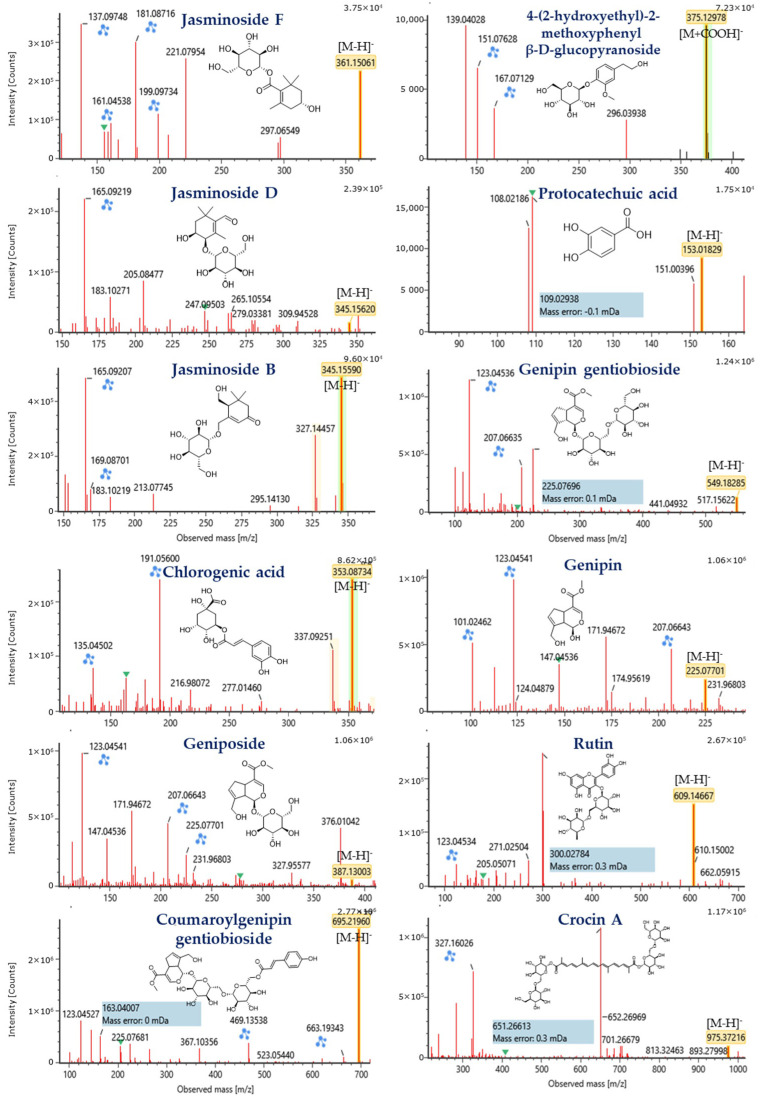
MS/MS spectrum of compounds identified from methanolic extract (MeOH-E) of *G. jasminoides* by LC-MS/MS analysis. Blue markings and the numbers in blue highlighter indicate the predicted MS/MS fragmentation of the compounds provided by MassFragment, an in silico fragmentation tool that uses a systematic bond disconnection approach to identify possible structures from the parent structure.

Flavonoids are a major group of molecules present in the plants with rich bioactivities including antioxidant, anti-diabetes, and anticancer properties. According to the earlier literature, a total of 22 flavonoids has been reported from the various extracts of *G. jasminoides* [14]. Similarly, the present study had identified compounds such as rutin (C_27_H_30_O_16_) and quercetin-3-O-β-d-glucopyranoside (C_21_H_20_O_12_) with MS/MS fragmentation of quercetin, aglycone of those two previously mentioned compounds, at *m*/*z* 300.0278 [M–rutinoside]^−^ and 300.0275 [M–Glc]^−^, respectively (Table 3, Figure 4).

##### Carotenoids

The carotenoids are a major constituent of the *G. jasminoides*, which is composed of carotenoids and similar compounds [61]. These compounds are used as food colorants as well as bioactive food additives. Based on the peaks observed from the LC-QTOF MS/MS analysis of MeOH-E, crocetin and crocin A were identified by their corresponding MS/MS fragmentats at *m*/*z* 283.1704 [M–COOH]^−^ for crocetin and 651.2661 [M–H–gentiobioside+H_2_O]^−^ and 327.1603 [M–H–gentiobioside ∗ 2+H_2_O ∗ 2]^−^ for crocin A (Table 3, Figure 4). Similarly, these compounds were reported from the flower and fruit of this plant [62,63].

##### Organic Acids and Others

According to the earlier research reports, a total of 30 organic acids with various bioactive properties, including phenolic acids and fatty acids can be isolated from *G. jasminoides* [14]. Similarly, the present study has identified a total of 13 organic acids and others from MeOH-E of *G. jasminoides* based on LC-MS/MS of deprotonated observed mass and its MS/MS fragmentation. The compounds were identified as chlorogenic acid (C_16_H_18_O_9_), caffeoylquinic acid (C_16_H_18_O_9_), dicaffeoylquinic acid (C_25_H_24_O_12_), protocatechuic acid (C_7_H_6_O_4_), quinic acid (C_7_H_12_O_6_), 2,4,6-trimethoxy-1-O-glucopyranoside (C_15_H_22_O_9_), 4-(2-hydroxyethyl)-2-methoxyphenyl β-d-glucopyranoside (C_15_H_22_O_8_), linolenic acid (C_18_H_30_O_2_), *n*-pentadecanal (C_15_H_30_O), linoleic acid (C_18_H_32_O_2_), acetylursolic acid (C_32_H_50_O_4_), palmitic acid (C_16_H_32_O_2_), and ethyl palmitate (C_18_H_34_O_2_). Further detailed identification information is shown in Table 3.

#### 2.6.2. Tentative Identification of the Compounds by GC-MS

GC-MS analysis evidenced the presence of fifteen volatile compounds classified into organic acids and their derivatives including fatty acids and phenolic acids in MeOH-E of *G. jasminoides* based on the electronic library, W8N05ST.L (Supplementary Table S1). The major compounds were found to be (9*Z*,12*Z*)-octadeca-9,12-dienoic acid (69.43%), hexadecanoic acid (16.09%), octadecanoic acid (8.32%), thymine (0.22%), 3,5-dihydroxy-6-methyl-2,3-dihydro-4*H*-pyran-4-one (0.26%), 3-carene (0.89%), 2-methylphenoxyacetic acid (0.49%), 2-amino-3-hydroxybenzoic acid (0.43%), 2,6-dimethyl-3-(methoxymethyl)-*p*-benzoquinone (0.60%), tetradecanoic acid (0.08%), methyl palmitate (0.20%), methyl linoleate (1.07%), methyl elaidate (0.55%), squalene (0.78%) and vitamin E (0.36%). Some of these compounds are known for promising antioxidant, antibacterial, and anticancer activities [64,65].

### 2.7. In Silico Screening of Enzyme Inhibitors

#### 2.7.1. Protein and Ligand Preparation

The protein and ligand were prepared according to the methods described earlier [12]. The protein molecular dock preparation was done using the AutoDock vina after the removal of the water molecules. Further, the ligand was selected for the molecular docking study based on Lipinski’s drug-likeness rules (Supplementary Table S2). The Lipinski’s indicated five rules, which is favor to select a compound as an orally active agent such as (i) the molecular weight of the compounds < 500 Da, (ii) hydrogen bond donor < 5, (iii) hydrogen bond acceptor < 10, (iv) miLogP < 5 and molar refractivity (40–130) [66]. Out of 33 unique compounds identified from MeOH-E of *G. jasminoides* by LC-MS/MS (Figure 3) and GC-MS (Supplementary Figure S2), a total of the 26 compounds were selected for the molecular docking study based on Lipinski’s rules satisfactory (Supplementary Table S2).

#### 2.7.2. Molecular Docking

##### Molecular Interaction with α-Amylase

**Figure 5:**
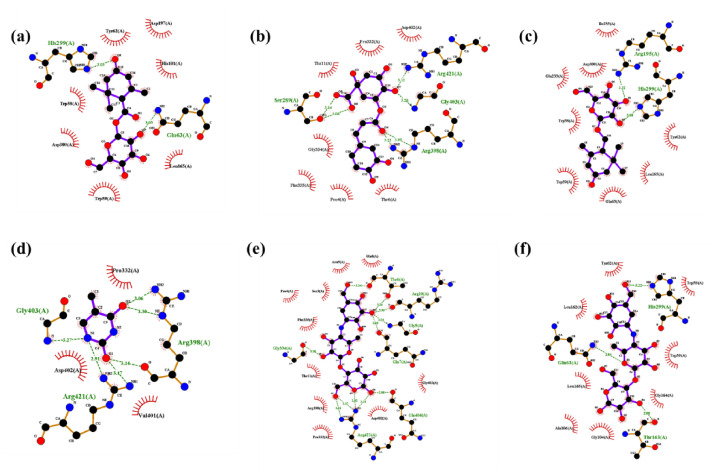
Molecular catalytic interaction of the compound identified from the methanolic extract (MeOH-E) of *G. jasminoides* with α amylase. Jasminoside F (**a**), chlorogenic acid (**b**), jasminoside A (**c**), thymine (**d**), acarbose derived trisaccharide (**e**), and acarbose (**f**) interacting with diabetes-related enzyme of α amylase (5E0F).

Molecular docking results revealed that all the selected compounds could interact with α-amylase. Among the compounds screened, jasminoside F, chlorogenic acid, jasminoside A, and thymine showed a higher docking score against α amylase (Table 4; Figure 5). The jasminoside F exhibited the binding affinity score of −8.5 kcal/mol with two hydrogen bond interactions with amino acid residues of His 299 and Gln63 in α amylase (Figure 5a). Chlorogenic acid showed the binding affinity score of -8.7 kcal/mol by interacting with amino acid residues of Arg421, Gly403, Arg398, Ser289 through six hydrogen bond interactions in α amylase (Figure 5b). Jasminoside A displayed a strong binding affinity score of −8.7 kcal/mol on α-amylase through interacting its amino acid residues of Arg195, His299 via two hydrogen bonds (Figure 5c).

The organic compound thymine showed a binding affinity score of −5.3 kcal/mol against α-amylase by interacting its residues of Gly403, Arg398, Arg421 by six hydrogen bonds (Figure 5d). Moreover, the positive control of the acarbose derived trisaccharide exhibited higher hydrogen bonds of 11 and amino acids (Thr6, Arg10, Gly9, Gln7, Gly334, Arg421, Gln404) interaction with α-amylase with binding affinity score of -8.3 kcal/mol (Figure 5e) while another control acarbose showed only three hydrogen bonds and amino acids (His299, gln63, Thr163) interactions with binding affinity score of −8.3 kcal/mol (Figure 5f). Overall, the results revealed that among the compounds tested, jasminoside A and chlorogenic acid were found to have the potential to interact with α-amylase with high binding affinity score than other molecules including positive controls. Similarly, the compound jasminoside is known for tyrosinase inhibition [56] while the phenolic compound chlorogenic acid exhibits anti-oxidative and anti-diabetic activities [67,68].

##### Molecular Interaction with α-Glucosidase

**Figure 6:**
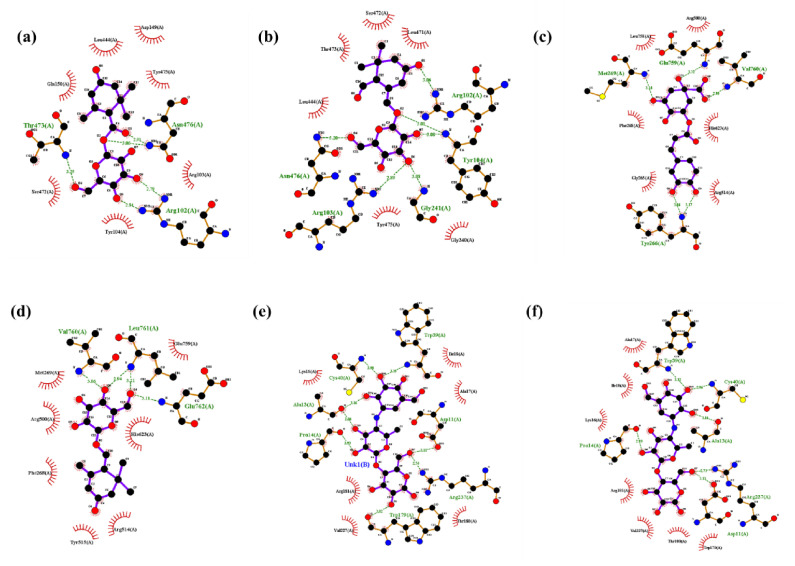
Molecular catalytic interaction of the compound identified from the methanolic extract (MeOH-E) of *G. jasminoides* with α glucosidase (5NN8). Jasminoside F (**a**), jasminoside B (**b**), chlorogenic acid (**c**), jasminoside A (**d**), acarbose derived trisaccharide (**e**), and acarbose (**f**) interacting with diabetes-related enzyme α glucosidase (5NN8).

The in silico docking study revealed that jasminoside F, jasminoside B, chlorogenic acid and jasminoside A displayed a higher binding affinity with α-glucosidase than other compounds studied in this study (Table 4; Figure 6). The interaction between jasminoside F and α-glucosidase showed the binding affinity score of −7.8 kcal/mol through the formation of five hydrogen bonds with amino acid residues such as Thr473, Asn476, Arg102 (Figure 6a). Jasminoside B established an interaction with α-glucosidase via six hydrogen bonds interacting with amino acids residues (Arg102, Tyr104, Gly241, Arg103, Asn476) of α-glucosidase with the binding affinity of 7.3 kcal/mol (Figure 6b). The chlorogenic acid exhibited the binding affinity score of −8.2 kcal/mol with five hydrogen bond interactions with amino acid residues of Met269, Glu759, Val760, Tyr266 in α-glucosidase (Figure 6c). The molecular interaction between jasminoside A and α-glucosidase exhibited a binding affinity of 7.8 kcal/mol by forming four hydrogen bonds with the amino acid residues Val760, Leu761, Glu762 (Figure 6d). However, the positive controls such as acarbose-derived trisaccharide and acarbose showed the promising dock binding affinity of 8.7 kcal/mol for interaction with α-glucosidase (Table 4). The acarbose-derived trisaccharide was found to establish an interaction with α-glucosidase through eight hydrogen bonds with amino acid residues of Trp39, Cys40, Ala13, Pro14, Asp11, Arg237, Trp179 (Figure 6e) while the acarbose established the interaction with α-glucosidase through six hydrogen bonds with amino acid residues of Trp39, Cys40, Pro14, Ala13, Arg237, Asp11 (Figure 6f). Overall, the docking study revealed that interactions with α-glucosidase of chlorogenic acid and jasminoside A were promising as compared to other compounds screened, and we hypothesize that these interactions might inhibit the activity of α-glucosidase. This finds the support of earlier works on the antidiabetic and enzyme inhibitory activities of these compounds [56,67,68].

## 3. Materials and Methods

### 3.1. Chemicals, Cell Line, and Maintenance

Ethidium bromide (EB), rhodamine 123 (Rh123), 2′-7′ dichlorofluorescein diacetate (DCFH-DA), acridine orange (AO), 2,2′-azinobis (3-ethylbenzothiazoline-6-sulfonic acid) diammonium salt (ABTS), 1, 2-diphenyl-1-picrylhydrazyl (DPPH), α-glucosidase, and α-amylase were purchased from Sigma-Aldrich (Seoul, Korea). The seed powder of *G. jasminoides* Ellis was procured from a local herbal company in South Korea, and authenticated by Professor M.H. Wang (Kangwon National University). Fetal bovine serum (FBS), penicillin and streptomycin, Dulbecco’s Modified Eagle Medium (DMEM), Roswell Park Memorial Institute Medium (RPMI) were obtained from ThermoFisher Scientific (Seoul, Korea). The cytotoxicity assay kit (WST-CELLO MAX™) was purchased from MediFab (Seoul, Korea), while the cell line human hepatic HepG2 cells and mouse fibroblast NIH3T3 cells were received from Korean Cell Line Bank, (KCLB, Seoul, Korea).

### 3.2. Preparation of Desiccative Ripe Fruits Extract

One hundred gram of seed powder (desiccative ripe fruits) of the *G. jasminoides* was extracted with methanol (1:5 ratio) for 24 h agitation in a magnetic stirrer. The methanol extract (MeOH-E) was filtered through Whatman no 1 filter paper and then concentrated using a rotary evaporator at 40 °C. Besides the water extraction was done according to the protocols described earlier [23]. The yield of MeOH-E and H2O-E was quantified using a weighing balance and then stored at 4 °C for further analytical experiments. The contents of total phenol and total flavonoids in MeOH-E were measured according to methods described earlier [69,70,71].

### 3.3. Antioxidant Activities

MeOH-E was analyzed for free radicals (DPPH and ABTS) scavenging activity according to the protocols reported earlier [72,73]. For DPPH inhibition assay, 100 µL of MeOH-E (1.95–1000 µg/mL) and 100 µL of DPPH (100 µM) were mixed and incubated at 27 °C for 10 min. Later the reaction mixture was observed at 517 nm using a UV spectrophotometer. The percentage of the DPPH scavenging was determined by adopting the formula reported earlier [74]. For the ABTS inhibition assay, firstly, the oxidative form of the ABTS^+^ was generated by mixing the potassium persulfate (2.45 mM) and ABTS (7 mM) at the ratio of the 0.5:1 ratio in dark conditions at 27 °C for 24 h. For the reaction, the 100 µL of ABTS^+^ and 100 µL of MeOH-E (1.95–1000 µg/mL ) were mixed and incubated at 27 °C for 10 min. Afterward, the reaction mixture was measured at 734 nm using a UV spectrophotometer. The percentage of ABTS scavenging = ((Control-sample)/control) × 100). The control is ABTS+solution alone.

### 3.4. Enzyme Inhibition Activities

The inhibition of α-glucosidase and α-amylase was measured according to previously reported methods [75,76,77]. Acarbose was used as a positive control for this experiment. For the α-glucosidase inhibition assay, 50 µL of MeOH-E (1.95–1000 µg/mL) was added to 20 µL of α-glucosidase (1 U) and this, 25 µL of *p*-nitrophenyl glucopyranoside (pNPG; 5 M) was added and incubated at 37 °C for 30 min. Later, the 100 µL of Na_2_ CO_3_ (0.1 M) was added to stop the reaction and measured at 405 nm using a UV spectrophotometer. For the α-amylase inhibition assay, 50 µL of MeOH-E (1.95–1000 µg/mL), 150 µL of starch (0.5%), 10 µL of α-amylase (2 U) were mixed and incubated at 37 °C for 30 min. Later 20 µL of NaOH (2 M) was added to stop the reaction. Then 20 µL DNS of (3,5-dinitrosalicylic acid) was added to the reaction solution and boiled for 20 min at 100 °C. Finally, the reaction mixture was cooled at room temperature and read at 540 nm using a UV spectrophotometer. The percentage of enzyme inhibition was determined by following the formula reported elsewhere [74].

### 3.5. Cell Culture Experiments

#### 3.5.1. Cytotoxicity

The cytotoxicity of MeOH-E was tested in the normal NIH3T3 cells and HepG2 cells (1 × 10^4^ cells/well) cultured in DMEM composed of FBS (10%), antibiotic solution (1%) for 24 h at 37 °C in 5% of a CO_2_ incubator. Later, the cells were treated with MeOH-E (0–100 µg/mL) for 24 h. After the treatment period, WST reagent (10 µL) was added, kept in a CO_2_ incubator for 1 h, and then OD was measured at 450 nm using UV spectrophotometer (SpectraMax^®^ Plus Microplate Reader, Molecular Devices, San Jose, CA, USA). The percentage of cell toxicity was calculated by adopting the formula reported previously [78].

#### 3.5.2. Determination of Glucose Uptake

To assess the MeOH-E induced glucose uptake in the HepG2 cells, an insulin-resistant model cell line (IR-HepG2) was firstly generated according to the protocol reported elsewhere [79,80]. The well-established IR-HepG2 cells (1 × 10^4^ cells/well) were cultured in high glucose DMEM incorporated with FBS (10%) and antibiotic solution (1%) in a 5% CO_2_ incubator for 24 h. For the treatment, various concentrations of MeOH-E (0–100 µg/mL) were added to cells and incubated for 24 h in the above-mentioned conditions. Besides, the positive control (HepG2) cells were maintained. After the incubation, the cells including the culture media were harvested and centrifuged at 440 g for 5 min, and the supernatant was used for glucose assay by DNS method. Glucose uptake (%) was estimated using the formula:(OD of high glucose DMEM media-IR-HepG2 cultured supernatant OD)/OD of high glucose DMEM media) × 100. Followed by the prevention of oxidative stress, mitochondrial membrane loss, and nucleus damage in IR-HepG2 by treatment of MeOH-E was observed using various staining assay as reported in earlier studies [81,82,83].

### 3.6. UHPLC-QTOF-MS/MS Analysis

For the UHPLC-QTOF-MS/MS analysis, MeOH-E was dissolved in 70% methanol, filtered with PTFE syringe filter (0.2 μm), and finalized in 20 ppm of MeOH-E. The LC/MS systems consisted of a Waters Acquity UPLC I-Class system (Waters Corp., Milford, MA, USA) coupled to Waters Xevo G2 QTOF mass spectrometer (Waters MS Technologies, Manchester, UK) equipped with an electrospray ionization (ESI) interface. The chromatographic separation was done with LC/MS equipped Waters Acquity UPLC BEH C18 (150 mm × 2.1 mm, 1.7 μm) (Waters Corp.). For the UHPLC, 2 µL of the sample was injected with a flow rate of 300 µL/min with a temperature of auto-sampler (10 °C) and column oven (40 °C). The mobile phases were 0.1% formic acid in H_2_O (A) and 0.1% formic acid in acetonitrile (B), and the following gradient was used: 10–90% B (0–12 min) and 100% B (12.1–16.0 min). The MS/MS data were obtained using a collision energy ramp from 15 to 45 eV in MS^E^ mode. The ESI parameters were set as follows: in negative ion mode in Continuum format, a capillary voltage of 2.5 kV, cone voltage of 45 V, source temperature of 120 °C, desolvation temperature of 350 °C, cone gas flow of 50 L/h, and desolvation gas flow of 800 L/h. The ion acquisition rate was 0.25 s with the mass range from *m*/*z* 100 to 1600. The instrument was calibrated using a sodium formate solution as the calibration standard. Leucine enkephalin (*m*/*z* 554.2615 in negative mode) was used as the reference lock mass at a concentration of 200 pg/μL and a flow rate of 5 μL/min and was sprayed into the MS instrument every 10 s to ensure accuracy and reproducibility. The data acquisition was measured by MassLynx V4.1 (Waters Corp.). The compounds were identified using the in-house phytochemical library (UNIFI 1.8; Waters Corp.) [40,41].

### 3.7. Gas Chromatography Analysis

The organic compounds present in MeOH-E was determined using a gas chromatography (Agilent 789A, Agilent, Santa Clara, CA, USA) mass spectrophotometry (Agilent 5975C; GC-MSD) system in the scan range of *m*/*z* 50–500 according to the detailed operation conditions described elsewhere [64,84]. The GS-MS used in this study was equipped with DB-5MS (30 m length × 0.25 mm inner diameter × 0.25 µm thickness of film) column and performed under operation condition as the flow rate of 1 mL/min, injection mode (5:1) with an inlet temperature of 250 °C, interface temperature of 280 °C, ion source of EI, 70 eV, with the temperature of 280 °C. The compounds present in the MeOH-E were tentatively identified by matching the GC-MS data with the electronic library of W8N05ST.L.

### 3.8. Molecular Docking

The compounds with enzyme inhibitory activity identified from MeOH-E were virtually analyzed against human lysosomal acid-α-glucosidase (PDB: 5NN8) and human pancreatic α-amylase (PDB: 5E0F) by molecular docking. The structure files of ligands were prepared using ChemBioDraw 15.0 (PerkinElmer, Waltham, MA, USA) and then saved as mol. These mol files of ligands were used for energy minimization according to the principle of gasteiger [85]. The 3D structure of PDB of 5NN8 and 5E0F were retrieved from RSCB (https://www.rcsb.org/) and before the docking experiment the water residue was removed and the binding packet size was prepared as reported earlier [12]. Finally, the molecular docking between various ligand and targeted protein was carried out using Autodock Vina 1.1.2. Finally, the interactions between the protein and compounds were observed using LIGPLOT+(v.2.2).

### 3.9. Statistical Analysis

All the experiments were executed in triplicate and the results are presented with mean ± standard error (SE). The descriptive statistics, student ‘*t*’ test, and analysis of various (ANOVA), line diagrams, Duncan’s multiple range test (DMRT) were made using excel. 2010 and SPSS (Ver 2016, IBM, Armonk, NY, USA). The difference at *p* < 0.05 was considered as significant among the factors.

## 4. Conclusions

In summary, this work analyzed the enzyme inhibition, anti-diabetic activities and metabolites present in the MeOH-E of *G. jasminoides* by using LC-MS/MS and GC-MS. The MeOH-E showed higher enzyme inhibition, antioxidant and anti-diabetic activities in IR-HepG2 cells. Metabolic profiling studies tentatively identified a total of 54 compounds including iridoids, terpenoids, fatty acid, phenolic acid derivatives from MeOH-E of *G. jasminoides* based on the observed *m*/*z* molecular ions in LC-MS/MS and GC-MS. The compounds identified were nine iridoid glycosides, 13 monoterpenoides, two each of flavonoids and carotenoids. Among the compounds identified chlorogenic acid and jasminoside A were found promising in interacting with α-glucosidase and α- amylase, as evidenced by molecular docking studies. Therefore, the present work concluded that bioactivity of the MeOH-E of *G. jasminoides* was the synergistic effect of various compounds present in the extract. According to the molecular screening, it is recommended that chlorogenic acid and jasminoside A be considered as candidate molecules for anti-diabetic activity. However, further studies are required for the purification and characterization of these two molecules and to determine their molecular mechanism of anti-diabetic activity for the development of future therapeutics.

## Figures and Tables

**Table 1:** Total Yield, Total Phenol, and Total Flavonoids Contents in Water (H2O-E) and Methanol Extracts (MeOH-E) of Seed Powder of the *G. jasminoides* Ellis.

Samples	Yield of the Extract (%)	Total Phenol (µg of TAE/g of Extract)	Total Flavonoids (µg of QE/g of Extract
MeOH-E	2.45 ^b^	769.47 ± 3.74 ^b^	487.54 ± 1.19 ^b^
H_2_O-E	1.58 ^a^	632.15 ± 1.25 ^a^	347.00 ± 2.49 ^a^

MeOH-E: Methanolic extract, H_2_O-E: Water extract, the results presented mean ±SE, tannic acid equivalent (TAE), quercetin equivalent (QE). The different superscript values indicated the significance among the type of extracts (*p* < 0.05).

**Table 2:** Antioxidant and Diabetes-Related Enzyme Inhibitory Activities of Water (H_2_O-E) and Methanol Extracts (MeOH-E) of Seed Powder of the *G. jasminoides.*

Samples	Inhibition Concentration (IC50:µg.mL^−1^)				Activity (µg Extract/µg AAEs)		Activity (µg Extract/µg ACEs)	
	ABTS Radical	DPPH Radical	α-Amylase Inhibition	α-Glucosidase Inhibition	ABTS Radical	DPPH Radical	α-Amylase Inhibition	α-Glucosidase Inhibition
MeOH-E	120.5 ± 1.09 ^a^	274.9 ± 1.42 ^a^	432.05 ± 0.51 ^a^	798.25 ± 0.84 ^a^	9.82 ± 0.62	6.42 ± 0.26	9.02 ± 0.25	6.52 ± 0.15
H_2_O-E	262.5 ± 0.18 ^b^	573.1 ± 0.85 ^b^	784.02 ± 0.88 ^b^	1052.23 ± 1.25 ^b^	13.20 ± 1.25	9.22 ± 0.81	15.22 ± 0.55	12.52 ± 0.61

MeOH-E: Methanolic extract, H_2_O-E: Water extract, the results presented mean ±SE, the different superscript in values indicated the significance among the type of extracts (*p* < 0.05). IC50 is indicated the concentration required to inhibit the 50% of free radicals or enzymes. ACEs: Acarbose equivalents, AAEs: ascorbic acid equivalents.

**Table 3:** Metabolite Profiling of Methanolic Extract (MeOH-E) of *G. jasminoides* by LC-MS/MS Analysis.

Component Name	RT (min)	Formula	Observed *m*/*z* [M–H]^−^	Mass Error (ppm)	Response	MS/MS Fragmentation (*m*/*z*)	References
Iridoids							
Geniposidic acid	1.04	C_16_H_22_O_10_	373.1142	0.4	6013	193.0507	[44,45]
Shanzhiside methyl ester	1.06	C_17_H_26_O_11_	405.1402	0.0	1994	229.0722, 391.1251	[45]
6β-Hydroxygeniposide	1.43	C_17_H_24_O_11_	403.1249	1.4	273,632	205.0511, 223.0615, 241.0721	[46]
Gardenoside	1.69	C_17_H_24_O_11_	403.1238	0.4	1146	207.0664, 225.0770	[45]
Genipin gentiobioside	1.70	C_23_H_34_O_15_	549.1828	0.4	464,390	207.0664, 225.0770	[45]
Genipin	2.00	C_11_H_14_O_5_	225.0770	0.2	81,760	193.0506, 207.0664	[44,46]
Geniposide	2.00	C_17_H_24_O_10_	387.1300	0.5	1,952,147	207.0664, 225.0770	[45,46]
Coumaroylgenipin gentiobioside	2.81	C_32_H_40_O_17_	695.2191	0.2	301,727	225.0768, 469.1354	[45,46]
Feruloylgenipin gentiobioside	2.89	C_33_H_42_O_18_	725.2300	0.2	108,903	193.0507, 225.0768	[47]
Monoterpenoides							
Jasminoside F isomers	1.25	C_16_H_26_O_9_	361.1506	0.5	36,284	137.0975, 181.0872, 199.0973	[45]
Jasminoside D	1.47	C_16_H_26_O_8_	345.1558	1.0	171,766	165.0922, 183.1027	[48]
Jasminoside B	1.66	C_16_H_26_O_8_	345.1599	0.1	37,456	151.0764, 165.0921, 169.0870	[46]
Jasminoside J	1.66	C_16_H_24_O_7_	327.1446	−1.1	2058	151.0764, 165.0921	[49]
Jasminodiol	1.96	C_10_H_16_O_3_	183.1029	0.2	2722	135.0817	[48]
Gardenate A	1.99	C_12_H_18_O_6_	257.1033	0.3	104	225.0770	[50]
Picrocrocinic acid	2.07	C_16_H_26_O_8_	345.1554	−0.1	47,270	165.0921	[45]
Jasminoside H	3.00	C_22_H_36_O_12_	491.2123	−0.3	30,153	167.1076, 323.0976	[46]
Crocusatin C	3.64	C_10_H_16_O_2_	167.1083	0.2	260	137.0973	[45,46,48]
Jasminoside A/E	3.67	C_16_H_26_O_7_	329.1606	0.1	3985	167.1079	[48]
6′-Sinapoyljasminoside C	3.92	C_27_H_34_O_11_	533.2021	−1.4	2024	165.0918, 205.0507	[48]
Methyl dihydrojasmonate	4.36	C_13_H_22_O_3_	225.1495	−0.5	241	181.1596	Pubchem
2-Hydroxyethylgardenamide A	6.69	C_13_H_17_NO_5_	266.1039	0.5	155	-	Pubchem
Flavonoids							
Rutin	2.28	C_27_H_30_O_16_	609.1464	0.3	16,687	300.0278	[47]
Quercetin-3-O-β-D-glucopyranoside	2.42	C_21_H_20_O_12_	463.0884	0.2	3003	300.027	[47]
Carotenoids							
Crocetin	2.67	C_20_H_24_O_4_	327.1589	−1.3	844	283.1704	[51]
Crocin A	3.95	C_44_H_64_O_24_	975.3707	−0.8	64,605	327.1603, 651.2661	[47,51]
Organic acids and others							
Quinic acid	0.81	C_7_H_12_O_6_	191.0563	1.2	85,121	137.0242, 173.0459	[52]
Trimethoxy-*O*-glucopyranoside	1.08	C_15_H_22_O_9_	391.1249	0.9	54,704	167.0716	Pubchem
4-(2-Hydroxyethyl)-2-methoxyphenyl β-d-glucopyranoside	1.37	C_15_H_22_O_8_	[M+COOH]^−^ 375.1298	0.3	2140	151.0763, 167.0713	Pubchem
Caffeoylquinic acid	1.44	C_16_H_18_O_9_	353.0876	−0.5	217	161.0248	[45]
Protocatechuic acid	1.51	C_7_H_6_O_4_	153.0193	−0.2	11,003	109.0294	[45]
Chlorogenic acid	1.70	C_16_H_18_O_9_	353.0878	0.0	4552	161.0248, 191.0562	[46,48]
Dicaffeoylquinic acid	2.79	C_25_H_24_O_12_	515.1196	0.2	6085	179.0350, 191.0559	[47]
Linolenic acid	10.53	C_18_H_30_O_2_	277.2174	0.2	1586	-	[53,54]
*n*-Pentadecanal	10.74	C_15_H_30_O	225.2217	−1.3	4866	-	[53]
Linoleic acid	11.60	C_18_H_32_O_2_	279.233	0.2	162,838	-	[54]
Acetylursolic acid	12.71	C_32_H_50_O_4_	497.3634	−0.5	3297	-	Pubchem
Palmitic acid	12.72	C_16_H_32_O_2_	255.2331	0.6	10,687	-	[53,54]
Ethyl palmitate	12.96	C_18_H_34_O_2_	281.2488	0.8	38,097	-	[53]

**Table 4:** Molecular Docking Analysis Catalytic Activity of Compounds Identified from the Methanolic Extract (MeOH-E) of *G. jasminoides* Against Diabetes Related Enzymes of α-Amylase and α-Glucosidase.

S.No	Compound	α-Amylase			α-Glucosidase		
		No. H Bonds	H Bond Interacting Amino Acids	Binding Affinity (kcal/mol)	No. H Bonds	H Bond Interacting Amino Acids	Binding Affinity (kcal/mol)
1	Quinic acid	3	Arg252	−5.7	4	His623, Leu761, Val760, Met269	−6.3
2	Jasminoside F	2	His 299, Gln63	−8.5	5	Thr473, Asn476, Arg102	−7.8
3	4-(2-Hydroxyethyl)-2-methoxyphenyl β-d-glucopyranoside	2	His299, Lys200	−6.9	2	Glu759, His490	−7.3
4	Jasminoside D	-	-	0	-	-	0
5	Protocatechuic acid	3	Arg421, Arg398	−5.5	2	Glu654, Ala655	−5.9
6	Jasminoside B	2	His299, Gln63	−7.7	6	Arg102, Tyr104,Gly241,Arg103, Asn476	−7.3
7	Jasminoside J	-	-	−8.1	2	Glu762, Leu761	−7.3
8	Chlorogenic acid	6	Arg421,Gly403,Arg398, Ser289	−8.7	5	Met269, Glu759, Val760, Tyr266	−8.2
9	Genipin	2	Arg195, His299	−6.6	1	Val760	−6.4
10	Crocusatin C	2	His305, Gln63	−5.9	3	Glu762, Met269, Leu761	−5.8
11	Jasminoside A	2	Arg195, His299	−8.7	4	Val760, Leu761, Glu762	−7.8
12	Thymine	6	Gly403, Arg398, Arg421	−5.3	3	Glu759, Ser757, Asp753	−5.1
13	3,5-Dihydroxy-6-methyl-2,3-dihydro-4*H*-pyran-4-one	4	Ala310, Gly309, Asn301, Arg346	−5.5	4	Arg317, Met314, Asn323	−5.1
14	3-Carene	-	-	−5.5	-	-	−5.3
15	2-Methylphenoxyacetic acid	1	Gln63	−5.6	3	Leu761, Val760, Glu759	−5.7
16	2-Amino-3-hydroxybenzoic acid	2	His299, Asp197	−5.6	5	Asn323, Leu311, Met314, Arg317	−5.3
17	2,6-Dimethyl-3-(methoxy-methyl)-*p*-benzoquinone	2	His185, Ala128	−5.5	2	Leu761, Met269	−5.5
18	Tetradecanoic acid	-	-	−5.8	-	-	−5.7
19	Methyl palmitate	2	His299, Asp197	−6.1	1	His301	−6.4
20	Hexadecanoic acid	-	-	−5.8	1	Glu759	−6.2
21	Methyl linoleate	1	Asp197	−6.5	1	Asn430	−6.3
22	Methyl elaidate	1	Asp197	−6.2	1	Asn430	−6.2
23	(9*Z*,12*Z*)-Octadeca-9,12-dienoic acid	3	Asn105, Ala106	−6.3	1	Arg491	−6.5
24	Octadecanoic acid	2	Asn105, Ala106	−6.2	1	His580	−6
25	Acarbose derived trisaccharide	11	Thr6, Arg10, Gly9, Gln7, Gly334, Arg421, Gln404	−8.3	8	Trp39, Cys40, Ala13, Pro14, Asp11, Arg237, Trp179	−8.7
26	Acarbose	3	His299, gln63, Thr163	−8.3	6	Trp39, Cys40, Pro14, Ala13, Arg237, Asp11	−8.7

## Data Availability

The data presented in this study are available on request.

## Supplementary Materials

The following are available online at https://www.mdpi.com/1424-8247/14/2/102/s1, Supplementary Figure S1. TIC chromatogram of metabolites profiling of methanolic extract (MeOH-E) of *G. jasminoides* by UHPLC-ESI-qTOF-MS/MS analysis. (a) Overall TIC chromatogram of MeOH-E of retention time (14 min) and (b) magnification of the TIC chromatogram from 1–5 min of retention time. Supplementary Figure S2. Low molecular weight and alkaloids identified from the methanolic extract(MeOH-E) of *G. jasminoides* by GCMS. Supplementary Table S1. GC-MS based analysis of alkaloids and low molecular weight molecules from methanolic extract (MeOH-E) of *G. jasminoides*. Supplementary Table S2. Assessment of the Drug-likeness through Lipinski’s strategies for methanolic extract (MeOH-E) of *G. jasminoides* by web tool (SwissADME).

## References

[1] Saeedi P., Petersohn I., Salpea P., Malanda B., Karuranga S., Unwin N., Colagiuri S., Guariguata L., Motala A.A., Ogurtsova K. (2019). Global and regional diabetes prevalence estimates for 2019 and projections for 2030 and 2045: Results from the International Diabetes Federation Diabetes Atlas, 9th edition. Diabetes Res. Clin. Pract..

[2] Behl T., Kotwani A. (2017). Anti-hyperglycemic effect of *Terminalia catappa* fruit extract in streptozotocin-induced diabetic rats. Int. J. Pharm. Pharm. Sci..

[3] Apoorva S.M., Sridhar N., Suchetha A. (2013). Prevalence and severity of periodontal disease in type 2 diabetes mellitus (non-insulin-dependent diabetes mellitus) patients in Bangalore city: An epidemiological study. J. Indian Soc. Periodontol..

[4] Ram Niwas J., Gyan Chand J. (2017). Evaluation of Antidiabetic Activity of Hydroalcoholic Extract of *Cassia fistula* Linn. pod in Streptozotocin-Induced Diabetic Rats. Pharmacogn. J..

[5] Fargion S., Dongiovanni P., Guzzo A., Colombo S., Valenti L., Fracanzani A.L. (2005). Iron and insulin resistance. Aliment. Pharmacol. Ther..

[6] Teng H., Yuan B., Gothai S., Arulselvan P., Song X., Chen L. (2018). Dietary triterpenes in the treatment of type 2 diabetes: To date. Trends Food Sci. Technol..

[7] Cade W.T. (2008). Diabetes-Related Microvascular and Macrovascular Diseases in the Physical Therapy Setting. Phys. Ther..

[8] Chawla R., Chawla A., Jaggi S. (2016). Microvasular and macrovascular complications in diabetes mellitus: Distinct or continuum?. Indian J. Endocrinol. Metab..

[9] Yu Z., Yin Y., Zhao W., Liu J., Chen F. (2012). Anti-diabetic activity peptides from albumin against α-glucosidase and α-amylase. Food Chem..

[10] Khanal P., Patil B.M. (2020). α-Glucosidase inhibitors from *Duranta repens* modulate p53 signaling pathway in diabetes mellitus. Adv. Tradit. Med..

[11] Wang P.-C., Zhao S., Yang B.-Y., Wang Q., Kuang H. (2016). Anti-diabetic polysaccharides from natural sources: A review. Carbohydr. Polym..

[12] Aispuro-Pérez A., López-Ávalos J., García-Páez F., Montes-Avila J., Picos-Corrales L.A., Ochoa-Terán A., Bastidas P., Montaño S., Calderón-Zamora L., Osuna-Martínez U. (2020). Synthesis and molecular docking studies of imines as α-glucosidase and α-amylase inhibitors. Bioorganic Chem..

[13] WHO (2019). Global Report on Traditional and Complementary Medicine.

[14] Chen L., Li M., Yang Z., Tao W., Wang P., Tian X., Li X., Wang W. (2020). *Gardenia jasminoides* Ellis: Ethnopharmacology, phytochemistry, and pharmacological and industrial applications of an important traditional Chinese medicine. J. Ethnopharmacol..

[15] Wang L., Yang C., Song F., Liu Z., Liu S. (2020). The therapeutic effectiveness of *Gardenia jasminoides* on type 2 diabetes rats: Mass spectrometry-based metabolomics approach. J. Agric. Food Chem..

[16] Stasiak N., Kukuła-Koch W., Głowniak K. (2014). Modern industrial and pharmacological applications of indigo dye and its de-rivatives—A review. Acta Pol. Pharm. Drug Res..

[17] Xiao W., Li S., Wang S., Ho C.-T. (2017). Chemistry and bioactivity of *Gardenia jasminoides*. J. Food Drug Anal..

[18] Chen J.-L., Shi B.-Y., Xiang H., Hou W.-J., Qin X.-M., Tian J.-S., Du G. (2015). 1H NMR-based metabolic profiling of liver in chronic unpredictable mild stress rats with genipin treatment. J. Pharm. Biomed. Anal..

[19] Wang G.-F., Wu S.-Y., Xu W., Jin H., Zhu Z.-G., Li Z.-H., Tian Y., Zhang J.-J., Rao J.-J., Wu S.-G. (2010). Geniposide inhibits high glucose-induced cell adhesion through the NF-κB signaling pathway in human umbilical vein endothelial cells. Acta Pharmacol. Sin..

[20] Pham T.Q., Cormier F., Farnworth E., Tong A.V.H., Van Calsteren M.-R. (2000). Antioxidant Properties of Crocin from *Gardenia jasminoides* Ellis and Study of the Reactions of Crocin with Linoleic Acid and Crocin with Oxygen. J. Agric. Food Chem..

[21] Higashino S., Sasaki Y., Giddings J.C., Hyodo K., Sakata S.F., Matsuda K., Horikawa Y., Yamamoto J. (2014). Crocetin, a Carotenoid from *Gardenia jasminoides* Ellis, Protects against Hypertension and Cerebral Thrombogenesis in Stroke-prone Spontaneously Hypertensive Rats. Phytother. Res..

[22] Dorman H., Peltoketo A., Hiltunen R., Tikkanen M. (2003). Characterisation of the antioxidant properties of de-odourised aqueous extracts from selected *Lamiaceae* herbs. Food Chem..

[23] Juma B.F., Majinda R.R.T. (2007). Constituents of *Gardenia volkensii*: Their brine shrimp lethality and DPPH radical scavenging properties. Nat. Prod. Res..

[24] Debnath T., Park P.-J., Nath N.C.D., Samad N.B., Park H.W., Lim B. (2011). Antioxidant activity of *Gardenia jasminoides* Ellis fruit extracts. Food Chem..

[25] Sayd S.S., Hanan A.A., Taie H.A.A., Taha L.S. (2010). Micropropagation, antioxidant activity, total phenolics and flavonoids con-tent of *Gardenia jasminoides* Ellis as affected by growth regulators. Int. J. Acad. Res..

[26] Gowd V., Bao T., Wang L., Huang Y., Chen S., Zheng X., Cui S., Chen W. (2018). Antioxidant and antidiabetic activity of blackberry after gastrointestinal digestion and human gut microbiota fermentation. Food Chem..

[27] Hua D., Luo W., Duan J., Jin D., Zhou X., Sun C., Wang Q., Shi C., Jiang Z., Wang R. (2018). Screening and identification of potent α-glycosidase inhibitors from *Gardenia jasminoides* Ellis. S. Afr. J. Bot..

[28] Saravana P.S., Cho Y.-N., Patil M.P., Cho Y.-J., Kim G.-D., Park Y.B., Woo H.-C., Chun B.-S. (2018). Hydrothermal degradation of seaweed polysaccharide: Characterization and biological activities. Food Chem..

[29] Hao S., Wang J., Li S., Shang F., Qin Y., Wu T., Bao X., Cao Q., Wang C., Sun B. (2020). Preparation of *Gardenia* red pigment and its antineoplastic activity in multiple tumor cells. Food Biosci..

[30] Moritome N., Kishi Y., Fujii S. (1999). Properties of red pigments prepared from geniposidic acid and amino acids. J. Sci. Food Agric..

[31] Saravanakumar K., Chelliah R., Shanmugam S., Varukattu N.B., Oh D.-H., Kathiresan K., Wang M.-H. (2018). Green synthesis and characterization of biologically active nanosilver from seed extract of *Gardenia jasminoides* Ellis. J. Photochem. Photobiol. B Biol..

[32] Wu X., Liu K., Liu P.-C., Liu R. (2015). Dual AO/EB Staining to Detect Apoptosis in Osteosarcoma Cells Compared with Flow Cytometry. Med. Sci. Monit. Basic Res..

[33] Zhang Q., Hu X.-F., Xin M.-M., Liu H.-B., Sun L., Morris-Natschke S.L., Chen Y., Lee K.-H. (2018). Antidiabetic potential of the ethyl acetate extract of Physalis alkekengi and chemical constituents identified by HPLC-ESI-QTOF-MS. J. Ethnopharmacol..

[34] Shao J., Xue J., Dai Y., Liu H., Chen N., Jia L., Huang J. (2012). Inhibition of human hepatocellular carcinoma HepG2 by phthalocyanine photosensitiser PHOTOCYANINE: ROS production, apoptosis, cell cycle arrest. Eur. J. Cancer.

[35] Ando T., Nagumo M., Ninomiya M., Tanaka K., Linhardt R.J., Koketsu M. (2018). Synthesis of coumarin derivatives and their cytoprotective effects on t -BHP-induced oxidative damage in HepG2 cells. Bioorganic Med. Chem. Lett..

[36] Song G., Sun Y., Liu Y., Wang X., Chen M., Miao F., Zhang W., Yu X., Jin J. (2014). Low molecular weight fluorescent probes with good photostability for imaging RNA-rich nucleolus and RNA in cytoplasm in living cells. Biomaterials.

[37] Zhang L., Mizumoto K., Sato N., Ogawa T., Kusumoto M., Niiyama H., Tanaka M. (1999). Quantitative determination of apoptotic death in cultured human pancreatic cancer cells by propidium iodide and digitonin. Cancer Lett..

[38] El Sayed A.M., Basam S.M., El-Naggar E.-M.B.A., Marzouk H.S., El-Hawary S. (2020). LC–MS/MS and GC–MS profiling as well as the antimicrobial effect of leaves of selected *Yucca* species introduced to Egypt. Sci. Rep..

[39] Kivilompolo M., Obůrka V., Hyötyläinen T. (2007). Comparison of GC–MS and LC–MS methods for the analysis of antioxidant phenolic acids in herbs. Anal. Bioanal. Chem..

[40] Yang Z.-R., Wang Z.-H., Tang J.-F., Yan Y., Yue S.-J., Feng W.-W., Shi Z.-Y., Meng X.-T., Peng C., Wang C.-Y. (2018). UPLC-QTOF/MSE and Bioassay Are Available Approaches for Identifying Quality Fluctuation of Xueshuantong Lyophilized Powder in Clinic. Front. Pharmacol..

[41] Jeong M.S., Park S., Han E.J., Park S.Y., Kim M.J., Jung K., Cho S.-H., Kim S.-Y., Yoon W.-J., Ahn G. (2020). *Pinus thunbergii* PARL leaf protects against alcohol-induced liver disease by enhancing antioxidant defense mechanism in BALB/c mice. J. Funct. Foods.

[42] Fu Z., Ling Y., Li Z., Chen M., Sun Z., Huang C. (2014). HPLC-Q-TOF-MS/MS for analysis of major chemical constituents of Yinchen-Zhizi herb pair extract. Biomed. Chromatogr..

[43] Hussain H., Green I.R., Saleem M., Raza M.L., Nazir M. (2019). Therapeutic Potential of Iridoid Derivatives: Patent Review. Inventions.

[44] Jia J., Liu M., Wen Q., He M., Ouyang H., Chen L., Li J., Feng Y., Zhong G., Yang S. (2019). Screening of anti-complement active ingredients from *Eucommia ulmoides* Oliv. branches and their metabolism in vivo based on UHPLC-Q-TOF/MS/MS. J. Chromatogr. B.

[45] Zhang S., Li Y., Zhang C.-X., Huang W.-Z., Ding G., Xiao W., Bi Y.-A., Xiao W. (2015). Research on the change of chemical composition in productive process of Re Du Ning Injections by HPLC/Q-TOF MS. Biomed. Chromatogr..

[46] Wu H., Li X., Yan X., An L., Luo K., Shao M., Jiang Y., Xie R., Feng F. (2015). An untargeted metabolomics-driven approach based on LC–TOF/MS and LC–MS/MS for the screening of xenobiotics and metabolites of Zhi-Zi-Da-Huang decoction in rat plasma. J. Pharm. Biomed. Anal..

[47] Wang L., Liu S., Xing J., Liu Z., Song F. (2016). Characterization of interaction property of multi-components in *Gardenia jasminoides* with aldose reductase by microdialysis combined with liquid chromatography coupled to mass spectrometry. Rapid Commun. Mass Spectrom..

[48] Wang L., Liu S., Zhang X., Xing J., Liu Z., Song F. (2016). A strategy for identification and structural characterization of compounds from *Gardenia jasminoides* by integrating macroporous resin column chromatography and liquid chromatography-tandem mass spectrometry combined with ion-mobility spectrometry. J. Chromatogr. A.

[49] Feng W., Dong Q., Liu M., Li S., Liu T., Wang X.-G., Niu L.-Y. (2017). Screening and identification of multiple constituents and their metabolites of Zhi-zi-chi decoction in rat urine and bile by ultra-high-performance liquid chromatography quadrupole time-of-flight mass spectrometry. Biomed. Chromatogr..

[50] Wang S.-C., Tseng T.-Y., Huang C.-M., Tsai T.-H. (2004). *Gardenia* herbal active constituents: Applicable separation procedures. J. Chromatogr. B.

[51] He W., Liu X., Xu H., Gong Y., Yuan F., Gao Y. (2010). On-line HPLC-ABTS screening and HPLC-DAD-MS/MS identification of free radical scavengers in *Gardenia* (*Gardenia jasminoides* Ellis) fruit extracts. Food Chem..

[52] Joo Y.H., Nam M.H., Chung N., Lee Y.K. (2020). UPLC-QTOF-MS/MS screening and identification of bioactive compounds in fresh, aged, and browned Magnolia denudata flower extracts. Food Res. Int..

[53] Wang C., Zhang N., Wang Z., Qi Z., Zhu H., Zheng B., Li P., Liu J. (2017). Nontargeted Metabolomic Analysis of Four Different Parts of *Platycodon grandiflorum* Grown in Northeast China. Molecules.

[54] Liu M., He M., Gao H., Guo S., Jia J., Ouyang H., Feng Y., Yang S. (2019). Strategy for rapid screening of antioxidant and anti-inflammatory active ingredients in *Gynura procumbens* (Lour.) Merr. based on UHPLC–Q-TOF–MS/MS and characteristic ion filtration. Biomed. Chromatogr..

[55] Breitmaier E. (2006). Hemi- and Monoterpenes. Terpenes: Flavors, Fragrances, Pharmaca, Pheromones.

[56] Chen Q.C., Youn U., Min B.-S., Bae K. (2008). Pyronane Monoterpenoids from the Fruit of *Gardenia jasminoides*. J. Nat. Prod..

[57] Yu Y., Xie Z.-L., Gao H., Ma W.-W., Dai Y., Wang Y., Zhong Y., Yao X.-S. (2009). Bioactive Iridoid Glucosides from the Fruit of *Gardenia jasminoides*. J. Nat. Prod..

[58] Akihisa T., Watanabe K., Yamamoto A., Zhang J., Matsumoto M., Fukatsu M. (2012). Melanogenesis Inhibitory Activity of Monoterpene Glycosides from *Gardeniae* Fructus. Chem. Biodivers..

[59] Peng K., Yang L., Zhao S., Chen L., Zhao F., Qiu F. (2013). Chemical constituents from the fruit of *Gardenia jasminoides* and their inhibitory effects on nitric oxide production. Bioorganic Med. Chem. Lett..

[60] Machida K., Oyama K., Ishii M., Kakuda R., Yaoita Y., Kikuchi M. (2000). Studies of the Constituents of *Gardenia* Species. II. Terpenoids from *Gardeniae* Fructus. Chem. Pharm. Bull..

[61] Chen Y., Yang Z.L., Zhang L.H., Liu S.J., Zhang X.T. (2011). Determination of geniposide, crocin and crocetin in different pro-cessing products of fructus *Gardeniae* by HPLC-ELSD. J. Chin. Med. Mater..

[62] Uekusa Y., Sugimoto N., Sato K., Yun Y.S., Kunugi A., Yamazaki T., Tanamoto K.-I. (2007). Neocrocin A: A novel crocetin glycoside with a unique system for binding sugars isolated from *Gardenia* yellow. Chem. Pharm. Bull..

[63] Cai L., Li R., Tang W.-J., Meng G., Hu X.-Y., Wu T.-N. (2015). Antidepressant-like effect of geniposide on chronic unpredictable mild stress-induced depressive rats by regulating the hypothalamus–pituitary–adrenal axis. Eur. Neuropsychopharmacol..

[64] Saravanakumar K., Chellia R., Hu X., Kathiresan K., Oh D.-H., Wang M.-H. (2019). Eradication of *Helicobacter pylori* through the inhibition of urease and peptide deformylase: Computational and biological studies. Microb. Pathog..

[65] Chandrasekaran M., Senthilkumar A., Venkatesalu V. (2011). Antibacterial and antifungal efficacy of fatty acid methyl esters from the leaves of *Sesuvium portulacastrum* L.. Eur. Rev. Med. Pharmacol. Sci..

[66] Lipinski C.A. (2004). Lead- and drug-like compounds: The rule-of-five revolution. Drug Discov. Today Technol..

[67] Ali N., Rashid S., Nafees S., Hasan S.K., Shahid A., Majed F., Sultana S. (2017). Protective effect of Chlorogenic acid against methotrexate induced oxidative stress, inflammation and apoptosis in rat liver: An experimental approach. Chem. Interact..

[68] Mccarty M.F. (2005). A chlorogenic acid-induced increase in GLP-1 production may mediate the impact of heavy coffee consumption on diabetes risk. Med. Hypotheses.

[69] Ardestani A., Yazdanparast R. (2007). Inhibitory effects of ethyl acetate extract of Teucrium polium on in vitro protein glycoxidation. Food Chem. Toxicol..

[70] Zhishen J., Mengcheng T., Jianming W. (1999). The determination of flavonoid contents in mulberry and their scavenging effects on superoxide radicals. Food Chem..

[71] Slinkard L., Singleton V.L. (1977). Total phenol analyses: Automation and comparison with manual methods. Am. J. Enol. Vitic..

[72] Blois M.S. (1958). Antioxidant Determinations by the Use of a Stable Free Radical. Nature.

[73] Cano A., Hernández-Ruíz J., García-Cánovas F., Acosta M., Arnao M.B. (1998). An end-point method for estimation of the total antioxidant activity in plant material. Phytochem. Anal..

[74] Sathiyaseelan A., Saravanakumar K., Mariadoss A.V.A., Wang M.-H. (2020). Biocompatible fungal chitosan encapsulated phytogenic silver nanoparticles enhanced antidiabetic, antioxidant and antibacterial activity. Int. J. Biol. Macromol..

[75] Kim Y.-M., Wang M.-H., Rhee H.-I. (2004). A novel α-glucosidase inhibitor from pine bark. Carbohydr. Res..

[76] Kandra L., Zajácz Á., Remenyik J., Gyémánt G. (2005). Kinetic investigation of a new inhibitor for human salivary α-amylase. Biochem. Biophys. Res. Commun..

[77] Saravanakumar K., Mariadoss A.V.A., Sathiyaseelan A., Wang M.-H. (2020). Synthesis and characterization of nano-chitosan capped gold nanoparticles with multifunctional bioactive properties. Int. J. Biol. Macromol..

[78] Saravanakumar K., Vivek R., Boopathy N.S., Yaqian L., Kathiresan K., Chen J. (2015). Anticancer potential of bioactive 16-methylheptadecanoic acid methyl ester derived from marine Trichoderma. J. Appl. Biomed..

[79] Chen L., Teng H., Cao H. (2019). Chlorogenic acid and caffeic acid from *Sonchus oleraceus* Linn synergistically attenuate insulin resistance and modulate glucose uptake in HepG2 cells. Food Chem. Toxicol..

[80] Teng H., Chen L., Song H. (2016). The potential beneficial effects of phenolic compounds isolated from *A. pilosa* Ledeb on insulin-resistant hepatic HepG2 cells. Food Funct..

[81] Saravanakumar K., Jeevithan E., Hu X., Chelliah R., Oh D.-H., Wang M.-H. (2020). Enhanced anti-lung carcinoma and anti-biofilm activity of fungal molecules mediated biogenic zinc oxide nanoparticles conjugated with β-D-glucan from barley. J. Photochem. Photobiol. B Biol..

[82] Saravanakumar K., Wang M.-H. (2019). Biogenic silver embedded magnesium oxide nanoparticles induce the cytotoxicity in human prostate cancer cells. Adv. Powder Technol..

[83] Sakthivel R., Malar D.S., Devi K.P. (2018). Phytol shows anti-angiogenic activity and induces apoptosis in A549 cells by depolarizing the mitochondrial membrane potential. Biomed. Pharmacother..

[84] Saravanan M., Senthilkumar P., Kalimuthu K., Chinnadurai V., Vasantharaj S., Ad P. (2018). Phytochemical and pharmacological profiling of *Turnera subulata* Sm., a vital medicinal herb. Ind. Crop. Prod..

[85] Wang J., Wang W., Kollman P.A., Case D.A. (2006). Automatic atom type and bond type perception in molecular mechanical calculations. J. Mol. Graph. Model..

