# Supplementary material for: Metabolite Profiling of Methanolic Extract of Gardenia jaminoides by LC-MS/MS and GC-MS and Its Anti-Diabetic, and Anti-Oxidant Activities
Source: Pharmaceuticals (Basel). 2021 Jan 28;14(2):102. doi: 10.3390/ph14020102 (PMC7912419; doi:10.3390/ph14020102)
Supplement: Supplementary file 1 [file pharmaceuticals-14-00102-s001.pdf]

(a)

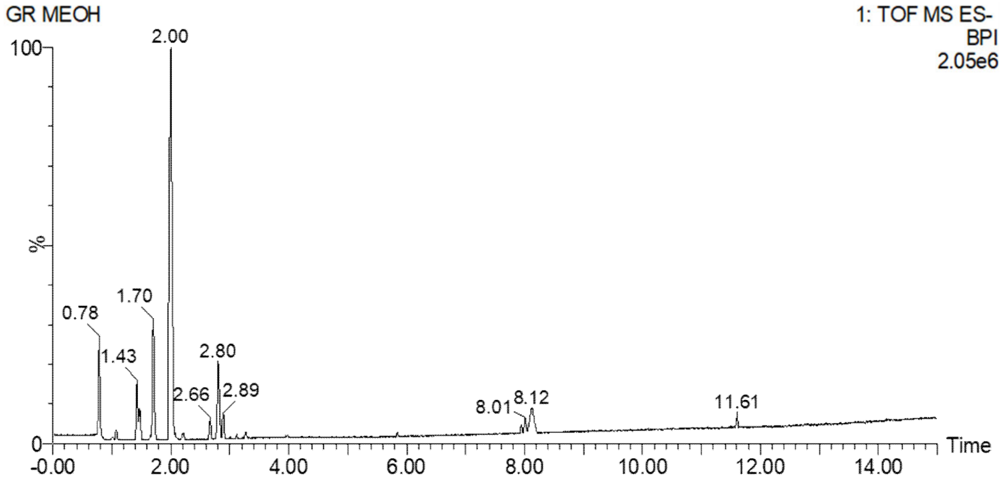

(b)

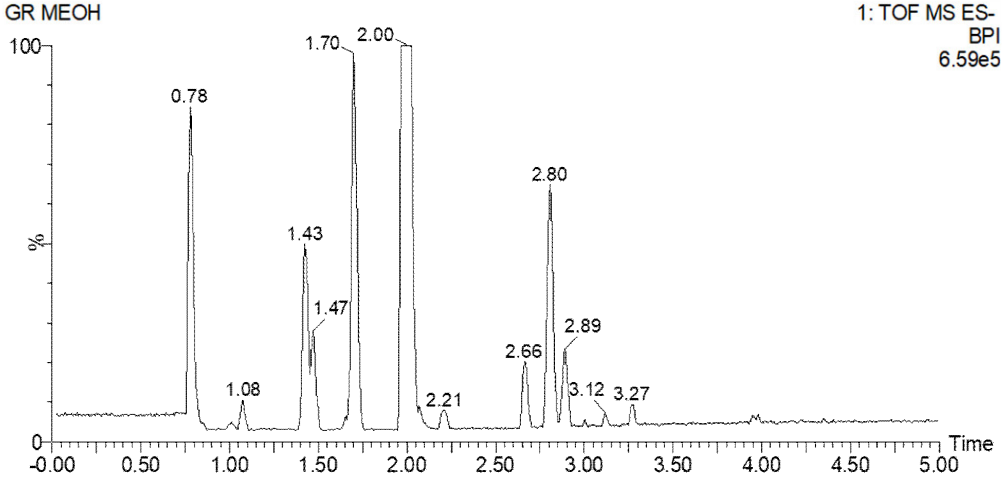

Supplementary Figure S1. TIC chromatogram of metabolites profiling of methanolic extract (MeOH-E) of *G. jasminoides* by UHPLC-ESI-qTOF-MS/MS analysis. (a) Overall TIC chromatogram of MeOH-E of retention time (14 min) and (b) magnification of the TIC chromatogram from 1-5 min of retention time.

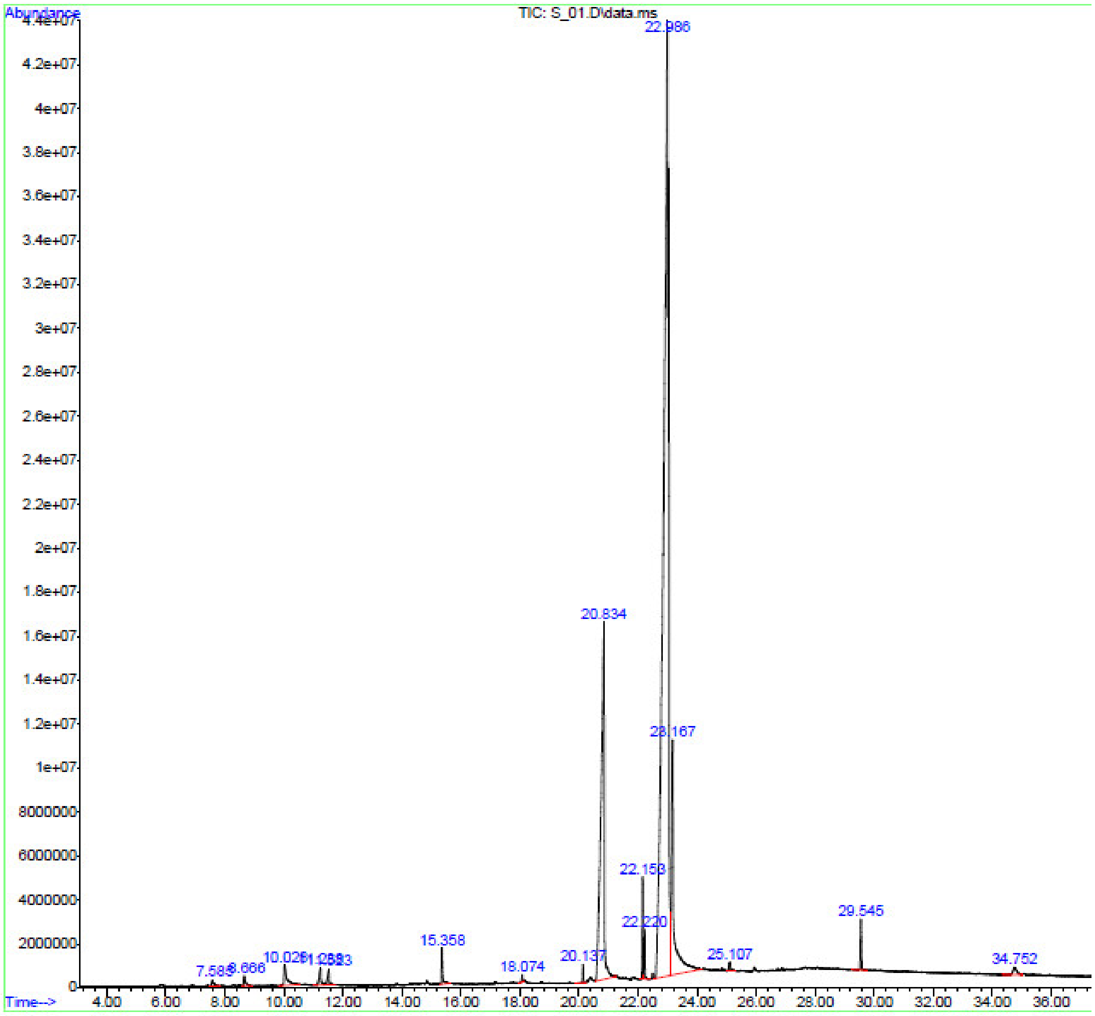

Supplementary Figure S2. Low molecular weight and alkaloids identified from the methanolic extract (MeOH-E) of *G.jasminoides* by GCMS.

Supplementary Table 1. GC-MS based analysis of alkaloids and low molecular weight molecules from methanolic extract (MeOH-E) of *G. jasminoides*.

Extract	IUPAC name of Compound	Retention time	Formula	Area (%)	Molecular weight (g/mol)
1	Thymine	7.586	$C_5H_6N_2O_2$	0.22	126.115
2	3,5-Dihydroxy-6-methyl-2,3-dihydro-4H-pyran-4-one	8.665	$C_6H_8O_4$	0.26	144.126
3	3-Carene	10.029	$C_{10}H_{16}$	0.89	136.238
4	2-Methylphenoxyacetic acid	11.238	$C_9H_{10}O_3$	0.49	166.176
5	2-Amino-3-hydroxybenzoic acid	11.524	$C_7H_7NO_3$	0.43	153.137
6	2,6-dimethyl-3-(methoxymethyl)-P-benzoquinone	15.358	$C_{10}H_{12}O_3$	0.60	180.203
7	tetradecanoic acid	18.072	$C_{14}H_{28}O_2$	0.08	228.376
8	Methyl palmitate	20.137	$C_{17}H_{34}O_2$	0.20	270.457
9	Hexadecanoic acid	20.832	$C_{16}H_{32}O_2$	16.09	256.43
10	Methyl linoleate	22.150	$C_{19}H_{34}O_2$	1.07	294.479
11	Methyl elaidate	22.223	$C_{19}H_{36}O_2$	0.55	296.495
12	(9Z,12Z)-octadeca-9,12-dienoic acid	22.985	$C_{18}H_{32}O_2$	69.43	280.452
13	Octadecanoic acid	23.167	$C_{18}H_{36}O_2$	8.32	284.484
14	Squalene	29.544	$C_{30}H_{50}$	0.78	410.73
15	Vitamin E	34.753	$C_{29}H_{50}O_2$	0.36	430.717

Supplementary Table 2. Assessment of the Druglikeness through Lipinski's strategies for methanolic extract (MeOH-E) of *G. jasminoides* by web tool (SwissADME).

Component name	Molecular weight (g/mol) <500Da	Number of Hydrogen bond		miLogP (<5)	Molar Refractivity (40-130)	Rules Satisfied
		Donor (<5)	Acceptor <10)			
Quinic acid	192.17	5	6	0.37	40.11	4/5
Jasminoside F	346.37	5	8	1.69	82.24	4/5
4-(2-Hydroxyethyl)-2-methoxyphenyl β-D-glucopyranoside	330.33	5	8	1.77	78.01	4/5
6β-Hydroxygeniposide	404.37	6	11	2.28	88.05	3/5
Jasminoside D	346.37	5	8	2.09	82.24	4/5
2,5-Dihydroxybenzoic acid	154.12	3	4	0.49	37.45	4/5
Jasminoside B	346.37	5	8	1.62	82.24	4/5
Jasminoside J	328.36	4	7	2.26	80.31	5/5
Chlorogenic acid	354.31	6	9	0.87	83.50	4/5
Genipin gentiobioside	550.51	8	15	2.89	119.28	2/5
Genipin	226.23	2	5	1.94	54.51	5/5
Geniposide	388.37	5	10	2.48	86.89	3/5
Crocin A	976.96	14	24	3.38	227.19	1/5
Dicaffeoylquinic acid	516.45	7	12	1.32	126.90	2/5
Coumaroylgenipin gentiobioside	682.62	9	17	2.09	156.33	2/5

Jasminoside H	492.51	7	12	2.36	113.46	3/5
Crocusatin C	168.23	1	2	1.92	48.70	5/5
Jasminoside A	330.37	4	7	2.30	81.08	5/5
Thymine	126.115	2	2	0.71	32.65	4/5
3,5-Dihydroxy-6-methyl-2,3-dihydro-4H-pyran-4-one	144.126	2	4	1.19	32.39	4/5
3-Carene	136.238	0	0	2.63	45.22	5/5
2-Methylphenoxyacetic acid	166.176	1	3	1.55	44.48	5/5
2-Amino-3-hydroxybenzoic acid	153.137	3	3	0.60	39.83	4/5
2,6-dimethyl-3-(methoxymethyl)-P-benzoquinone	180.203	0	3	1.93	48.61	5/5
Tetradecanoic acid	228.376	1	2	3.32	71.18	5/5
Methyl palmitate	270.45	0	2	4.41	85.12	5/5
Hexadecanoic acid	256.42	1	2	3.85	80.80	5/5
Methyl linoleate	294.47	0	2	4.61	93.78	5/5
Methyl elaidate	296.49	0	2	4.75	94.26	5/5
(9Z,12Z)-octadeca-9,12-dienoic acid	280.45	1	2	4.14	89.46	5/5
Octadecanoic acid	284.48	1	2	4.30	90.41	5/5
Squalene	410.72	0	0	6.37	143.48	3/5
Vitamin E	430.71	1	2	5.92	136.27	3/5
